# Detection of Glaze Icing Load and Temperature of Composite Insulators Using Fiber Bragg Grating

**DOI:** 10.3390/s19061321

**Published:** 2019-03-16

**Authors:** Jie Wei, Yanpeng Hao, Yuan Fu, Lin Yang, Jiulin Gan, Zhongmin Yang

**Affiliations:** 1 School of Electric Power, South China University of Technology, Guangzhou 510640, China; epjie1123@mail.scut.edu.cn (J.W.); yphao@scut.edu.cn (Y.H.); epyuanfu@mail.scut.edu.cn (Y.F.); 2 State Key Laboratory of Luminescent Materials and Devices, South China University of Technology, Guangzhou 510640, China; msgan@scut.edu.cn (J.G.); yangzm@scut.edu.cn (Z.Y.)

**Keywords:** glaze icing detection, fiber Bragg grating (FBG), composite insulator with embedded FBG, simultaneous measurement of temperature and strain

## Abstract

Conventional methods for the online monitoring of icing conditions of composite insulators suffer from difficulties. To solve this issue, a novel method is first proposed to detect glaze icing load via embedding three optical fibers with fiber Bragg gratings (FBGs) into a 10 kV composite insulator. Specifically, FBG temperature compensation sensors were packaged in ceramic tubes to solve strain and temperature cross-sensitivity. Temperature effect experiments and simulated glaze icing load experiments were performed to verify the feasibility of the proposed method. The results show that temperature sensitivities of all FBGs are identical (i.e., 10.68 pm/°C), which achieves a simultaneous measurement of temperature and strain. In addition, the proposed method can detect glaze icing load of the composite insulator above 0.5 N (i.e., 15% of icicle bridged degree) in the laboratory.

## 1. Introduction

Since the 21st century, the global climate has changed dramatically, and the shortage of conventional energy has become increasingly serious. Meanwhile, economic development and social progress make pressing demands for the safe operation and development of power grids [1]. Smart grid construction has become a trend to address future challenges for the electric power industry [2]. An essential part of the smart grid is smart sensing technology, which can sensitively and accurately monitor most of electrical equipment. Additionally, the monitor signal can be reliably and safely transmitted back in harsh environments [1,2].

The transmission lines often cross different kinds of terrain and complex climate areas. Therefore, the transmission lines have the characteristics of large scatter, long distance, and comprehensive coverage. Especially in China, insulators of the transmission lines are easily covered by contamination and ice in cold and high humidity regions, which often causes flashover accidents and even severe disasters of the power grid [3]. Thus, monitoring transmission lines is an important part of smart grid construction, especially for insulators that undertake the insulation and mechanical support performance [2,3,4].

In the last decade, many researchers have proposed methods to access and monitor the contamination of insulators. For example, Ekonomou [5,6,7] proposed approaches to access the contamination based on artificial neural networks (ANN) and a multi-model partitioning filter (MMPF). Cai proposed a method to monitor the contamination of insulators online using optical technology [8]. However, there are no appropriate methods to monitor icing conditions of insulators.

With the development of computer graphics, some researchers have proposed methods for detecting icing conditions of insulators based on video or image processing. To date, the conventional detection methods are generally classified into three types, i.e., ultraviolet imaging methods, unmanned aerial vehicle (UAV) detection methods, and video camera detection methods.

(1) Ultraviolet imaging methods for monitoring insulators icing flashover

When icing and pollution flashover of insulators occurs, the arc causes partial carbide passages to be semi-conductive; also referred to as the tracking phenomenon [9]. The insulator performance can be estimated using an ultraviolet imager to observe ultraviolet photon number, facula area, and facula number [10]. However, this method is affected by illumination conditions in the environment and ultraviolet from the corona of grading rings. Moreover, some insulator defects can only cause partial flashover after wetting, which is difficult to monitor using the ultraviolet imaging method.

(2) UAV detection methods for monitoring icing insulators

In recent years, unmanned aerial vehicles carrying cameras have been widely used to monitor insulators of transmission lines. However, these methods are easily affected by harsh weather and environmental visibility [11]. Specifically, Wu proposed a texture segmentation algorithm to process the complex aerial insulator images, which could detect the icing condition of insulators [12]. However, this method is affected by environmental visibility, causing blurry images. In addition, unmanned aerial vehicles cannot fly in heavy rain and intense wind weather and, consequently, they are not suitable for monitoring insulator conditions in all weather.

(3) Video camera detection methods for monitoring icing insulators

Video cameras are installed on transmission line towers to take pictures of icing insulators and conductors, and then the pictures are transmitted by a GPRS network. Finally, the icing conditions of insulators are calculated by image processing methods. However, these methods are limited by the harsh environment and field power. For example, Berlijn proposed a method to detect the ice and snow performance of insulators based on threshold images [13]. Hao proposed graphical shed spacing and graphical shed overhang to assess icing condition of insulators based on a GrabCut segmentation algorithm [14]. In harsh environments, the video cameras are covered by ice, causing similar blurry images as those obtained using UAV detection methods. Even worse, because they are limited by field power, the cameras may fail after consecutive ice days, which are not suitable for long-term monitoring.

Compared with the conventional methods that are limited by environmental condition, field power, and strong electromagnetic field, optical fiber sensors (OFS) are small in size, immune to electromagnetic interference, chemically resistant, and have no power supply requirement. Thus, these advantages make OFS well suited for sensing electrical equipment under harsh conditions. To date, OFS have been successfully developed for the electric power industry [15,16,17,18,19]. Fiber Bragg grating (FBG) sensors are one of the typical OFS. By demodulating wavelength signal, the FBG can measure many characteristic values of objects, such as strain/stress, temperature, wind speed/direction, vibration, and others. In addition, FBG is better suited for embedding into the composite insulator and monitor icing condition because the composite insulator is long and stick-shaped, which can help in the online monitoring of power grid operating conditions on a large scale [19]. Moreover, FBG can also monitor the operating conditions of composite insulators, including heating, brittle/decay-like fracture, and partial flashover in the future.

To date, many researchers have contributed to the study of the composite insulator with embedded FBG [19,20,21,22,23,24,25,26,27,28,29,30,31]. In 1992, Japanese scientist Seike [20] proposed embedding optical fiber into the composite insulator for monitoring its internal stress. In 2003 and 2004, Lepley and Trouillet [21,22] presented a design where the optical fiber was twisted onto the insulator core rod. When the internal temperature of the insulator was so high that the optical fiber coating was melted locally, the interrogator could not receive optical signals and diagnose faults. However, this method diagnoses faults after they occur, and cannot monitor the degradation process of the insulator core rod. Kumosa [23,24] and Portnov [25] researched the relationship between the wavelength of FBG embedded into the core rod and the internal stress via finite element analysis and tension test. China Electric Power Research Institute [26,27,28,29] calculated the relationship between the FBG wavelength shift and stress distribution of the core rod via finite element analysis and the transfer matrix method. The results showed that FBG wavelength shift was linearly related to the axial stress of the core rod and environmental temperature. In 2012 and 2017, Chen [30,31] embedded FBG into the fiber-reinforced polymer (FRP) rod of the composite insulator to monitor its internal temperature, and then established a theoretical model. 

The above papers have verified the feasibility of embedding FBG sensors into composite insulators. However, the methods embedded the optical fiber into the core rod, which was not suitable for detecting icing load on the composite insulator sheds. In addition, the methods used electric temperature sensors to compensate for the FBG wavelength shift caused by temperature changes. However, the electric temperature sensors were affected by strong electromagnetic fields and chemical corrosion.

In summary, the main contribution of the paper can be summarized as follows. First, a novel method is proposed that can detect glaze icing load and temperature of composite insulators via embedding three FBG arrays into the composite insulator. In order to solve strain and temperature cross-sensitivity, the FBG temperature compensation sensors were packaged into ceramic tubes for insulation. Then, the arrangement of FBGs in the composite insulator is described in detail, and experiments were performed to verify the performance of the composite insulator with embedded FBG. After that, the relationships among FBG wavelength shifts, temperature, and the simulated glaze icing load are analyzed respectively. Finally, experimental results indicate the FBG strain sensor can detect the glaze icing load of the composite insulator in the laboratory.

## 2. Detection Principle

**Figure 1:**
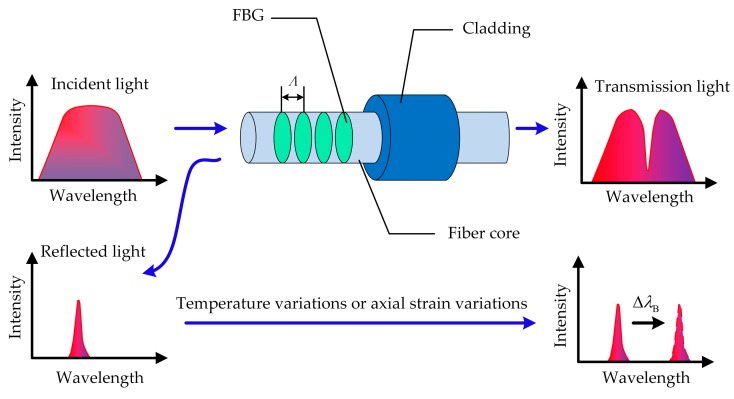
Schematic diagram of the fiber Bragg grating (FBG) sensing principle.

The optical fiber is the abbreviation of fiber waveguide or optical waveguide fiber whose structure is the coaxial cylinder. Typically, the optical fiber includes the fiber core, cladding, and coating from inside to outside, as shown in Figure 1. The kernels of an optical fiber are the fiber core and cladding, which are the primary channel of transmission light. In addition, the coating can protect the optical fiber from moisture and mechanical abrasion, which helps increase the flexibility and lifetime of the optical fiber. 

### 2.1. FBG Sensing Principle

Fiber Bragg grating (FBG) is a periodic modulation of the index of refraction in the core of an optical fiber. When a broadband light transmits along the fiber core, the FBG reflects a narrow band portion with a specific wavelength, whereas the rest portion of the broadband light passes. Since the external environment (e.g., temperature and strain) can change the periodic modulation of the index of refraction, the center wavelength of the reflected spectrum changes with temperature and strain. Therefore, the temperature and strain of objects can be detected via wavelength shifts of FBGs [32]. The FBG reflective center wavelength is defined as *λ*_B_:

(1) $$\lambda_{B}=2n_{\mathrm{eff}}\cdot \Lambda ,$$

where *n*_eff_ is the effective refractive index of the optical fiber, and Λ denotes the periodicity of the grating.

The wavelength shift is decided by the changes of *n*_eff_ and Λ, which are mainly affected by environmental temperature variations (Δ*T*) and axial strain variations (Δ*ε*). In this case, the FBG wavelength shift can be described as [33]

(2) $$\begin{array}{c} \Delta \lambda_{B}=\lambda_{B}(\alpha_{f}+\xi )\Delta T+\lambda_{B}(1-P_{e})\Delta \varepsilon \\ =K_{B}{}_{,T}\Delta T+K_{B}{}_{,\varepsilon }\Delta \varepsilon \end{array},$$

where *α_f_* denotes the thermal expansion coefficient, *ξ* denotes the thermo-optic coefficient, *P_e_* denotes the elastic-optic coefficient, and *K*_B,*T*_ and *K*_B,*ε*_ denote the temperature sensitivity (pm/°C) and the axial strain sensitivity (pm/με), respectively. Since *K*_B,*T*_ and *K*_B,*ε*_ are both constant as determined by temperature and the initial stress state, the center wavelength shifts of FBG are linearly related to temperature and axial strain, respectively.

### 2.2. Simultaneous Measurement of FBG Temperature and Strain 

As indicated in Equation (2), the FBG center wavelength shift is decided by the temperature and axial strain. In addition, while ice is covering an insulator shed, the temperature and stain of insulator sheds are changing with glaze icing load. Therefore, it is necessary to simultaneously measure temperature and strain of insulator sheds when the wavelength shifts are demodulated. A proposed method for temperature compensation is used to measure FBG temperature and strain simultaneously. More specifically, two FBGs (i.e., FBG-1 and FBG-2) are recorded on a fiber core. FBG-1, with a specific center wavelength, is used to measure temperature, and it is not affected by strain, as shown in Equation (3). FBG-2, with the other specific center wavelength, is used to measure temperature and strain, and it is affected by temperature and strain, as shown in Equation (4). In laboratory experiments, there are no obvious non-uniform temperature distributions during glaze ice accretion on composite insulators. Thus, the temperature of FBG strain sensors could be considered equivalent to that of FBG temperature compensation sensors. The wavelength shifts of two FBGs caused by temperature are both Δ*λ*_B1_, and the wavelength shift of FBG-2 caused by strain is Δ*λ*_B2_ − Δ*λ*_B1_. As above, the simultaneous measurement for FBG temperature and strain is overcome via the temperature compensation method.

(3) $$\Delta \lambda_{B1}=K_{B}{}_{1,T}\Delta T$$

(4) $$\Delta \lambda_{B2}=(K_{B}{}_{2,T}\Delta T+K_{B2}{}_{,\varepsilon }\varepsilon_{2})$$

(5) $$\Delta \lambda_{compensation}=\Delta \lambda_{B2}-\Delta \lambda_{B1}$$

## 3. Design of the Composite Insulator with Embedded FBG, and Experiments

### 3.1. Fiber Bragg Grating Parameters and Packing

In this paper, four FBGs were written in every single mode optical fiber via the phase mask method. Their parameters are indicated in Table 1.

**Figure 2:**
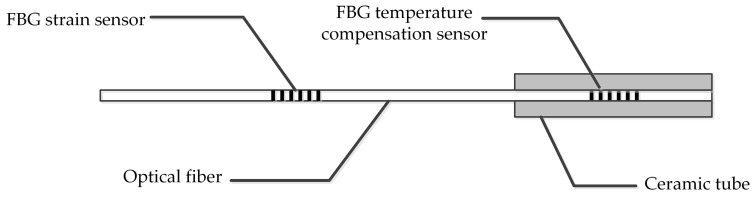
The FBG packing method.

The FBG temperature compensation sensor is packed in a ceramic tube for insulation. The ceramic tube is cylindrical, with a length of 30 mm and diameter of about 2 mm, as shown in Figure 2. By using a ceramic tube to pack the FBG, FBG temperature compensation sensors are only affected by temperature, and meet the insulation performance.

Three optical fibers were embedded into a composite insulator, which were labeled as 1# Optical Fiber, 2# Optical Fiber, and 3# Optical Fiber, respectively. To compare the mechanical behavior from insulator sheds to FBGs, the FBG strain sensors in 1# Optical Fiber and 2# Optical Fiber were bare, whereas 3# Optical Fiber was packed in a PVC protection sleeve whose diameter was 0.9 mm.

### 3.2. Design of the Composite Insulator with Embedded FBG

**Figure 3:**
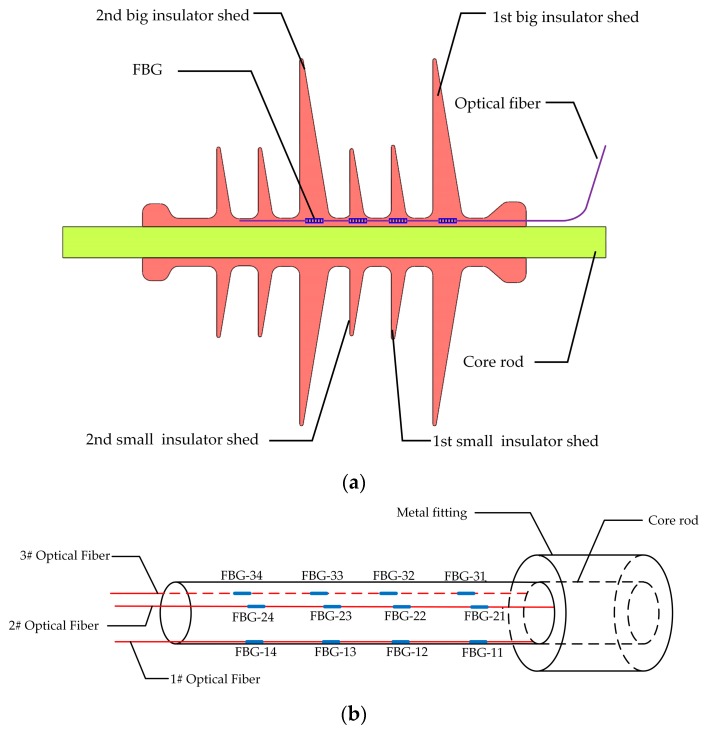

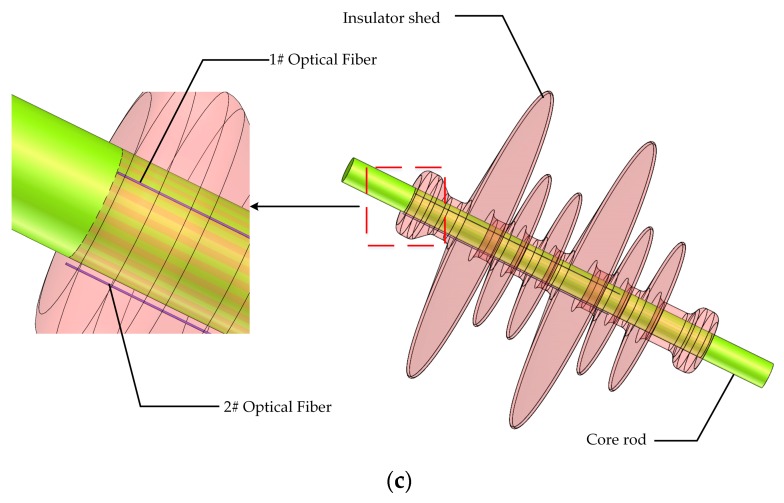
The diagram of optical fiber embedded position distribution. (**a**) Relative positions between FBGs and insulator sheds; (**b**) FBG distribution on the composite insulator, (**c**) Relative positions between FBGs and composite insulator.

The composite insulator model is FXBW-10/70, whose parameters are indicated in Table 2. Figure 3 depicts the design of a composite insulator with embedded FBG. Three optical fibers were embedded vertically into the composite insulator with even distribution, and they were separated at 120° intervals around the central axis of the composite insulator. Furthermore, four FBGs were written in every optical fiber, which were labeled as FBG-*mn*, where *m* was the label of the optical fiber and *n* was the label of the optical fiber grating. For example, four FBGs in the 1# Optical Fiber were separately labeled as FBG-11, FBG-12, FBG-13, and FBG-14, where FBG-11, FBG-12, and FBG-13 were all FBG strain sensors, and FBG-14 was the FBG temperature compensation sensor. These sensors were respectively located in the roots of the 1st big insulator shed, the 1st small insulator shed, the 2nd small insulator shed, and the 2nd big insulator shed, as depicted in Figure 3a. In the same way, FBG-21, FBG-22, and FBG-23 in 2# Optical Fiber and FBG-31, FBG-32, and FBG-33 in 3# Optical Fiber were all FBG strain sensors. FBG-24 and FBG-34 were both FBG temperature compensation sensors, as depicted in Figure 3b. The initial center wavelengths of all FBGs are shown in Table 3.

### 3.3. The Detection System of the Composite Insulator with Embedded FBG

**Figure 4:**
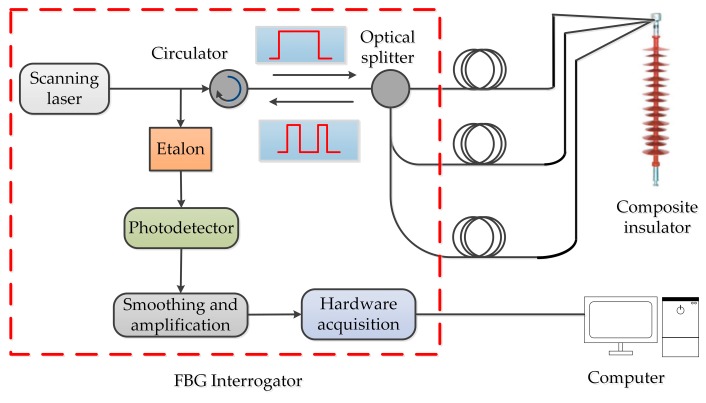
The system diagram of glaze icing detection of a composite insulator with embedded FBG.

The detection system mainly includes two parts, which are a composite insulator with embedded FBG and an FBG interrogator, as depicted in Figure 4. The type of interrogator was CASSTK-SA, whose detection wavelength range is from 1525 to 1565 nm, and the wavelength interrogator accuracy is 2.5 pm, the temperature detection accuracy is 0.5 °C, and the stress detection accuracy is 0.5% F.S. Additionally, the interrogator can detect eight channels (i.e., eight optical fibers) at the same time where each channel can detect forty FBGs at most. In experiments, the FC/APC connectors are inserted into the interrogator, and FBG wavelength shifts are shown on the computer.

To verify the feasibility of detecting glaze icing load via composite insulators with embedded FBG, temperature effect experiments and simulated glaze icing load experiments were performed via the detection system.

### 3.4. Temperature Effect Experiments

The temperature effect experiments were performed in order to research the relationship between FBG wavelength shifts and temperature. In natural conditions, the insulators are easily covered by glaze ice at a temperature between −8 and 0 °C [34]. When the environmental temperature is below −10 °C, the glaze ice on insulators surface stops growing because of the rapidly decreasing water content of air [35]. Furthermore, IEEE Standard 1783-2009 recommended that the environmental temperature is from −15 to −5 °C for simulating glaze ice accretion on insulators surface in the climate room [36]. Therefore, the temperature effect experiments were performed within the range of −15 to +50 °C, and the sampling interval was 5 °C. Before the experiments, the composite insulator with embedded FBG was placed in a thermostat, which was used to control the surrounding temperature. The specific experimental process was shown as follows:

(1) A composite insulator with embedded FBG was placed in the thermostat, and the optical fiber connectors were inserted into the interrogator. Then, when the temperature was +20 °C, the wavelengths of all FBGs were recorded as initial wavelengths.

(2) The temperature range of thermostat was set from −15 to +50 °C, and the wavelength shifts were recorded at intervals of 5 °C. Note that it took about 8 h when external and internal temperatures of the composite insulator were consistent [28]. After placing the composite insulator into a thermostat for 8 h, the temperature of the composite insulator could be considered as a constant and recorded if the wavelength shift was less than 2.5 pm/min [28]. After averaging many sets of experimental data, the results of temperature effect experiments were created.

### 3.5. Simulated Glaze Icing Load Experiments

In order to verify the method feasibility, simulated glaze icing load experiments were performed to research the relationship between simulated glaze icing load and wavelength shifts.

**Figure 5:**
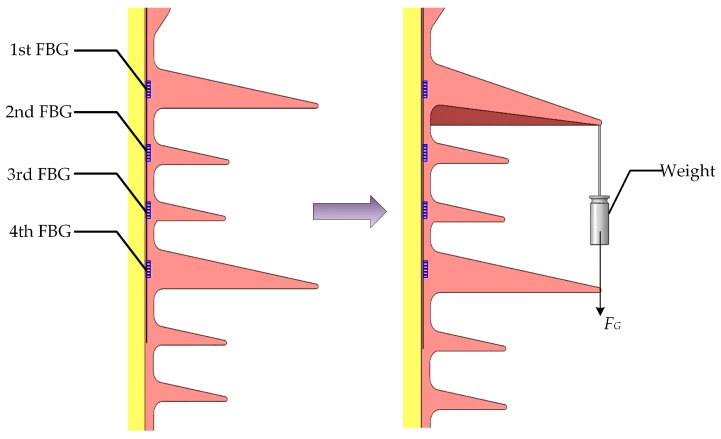
The experiment diagram of hanging a weight on an insulator shed for simulating glaze icing load.

Many studies have indicated that the non-uniform glaze ice accretion on insulators is common, which causes the highest probability of flashover occurrence [37,38]. Therefore, in this experiment, a weight was hung on each insulator shed for simulating glaze icing load generated by an icicle (i.e., non-uniform glaze ice), as depicted in Figure 5 [39]. For simulating glaze icing conditions of the composite insulator, icicle length and icicle bridged degree were proposed, which can be described as [40,41,42]

(6) $$\eta =\frac{S-d}{S}=\frac{L}{S},$$

where *η* denotes the icicle bridged degree along the insulator, *S* is the shed spacing between big sheds, *d* is the gap length, and *L* is icicle length.

The experimental process is described as follows:

(1) A composite insulator with embedded FBG was hung on a steel tower, and optical fiber pigtails were inserted into the interrogator. Then, the interrogator was turned on and the initial wavelengths of all FBGs were recorded.

(2) In order to simulate the non-uniform glaze icing load generated by an icicle with typical geometry proposed in [40], the weights were set as 0.5, 1.0, 1.5, 2.0, and 2.5 N. Furthermore, according to the geometry of the icicle [40], the weights can correspond to icicle lengths and icicle bridged degrees (Table 4), which are common parameters in the icicle growth experiments [40,41,42].

(3) A weight was hung on each insulator shed edge facing an FBG strain sensor. If the wavelength shift was less than 2.5 pm/min within 3 min, the stress state of composite insulator could be considered as stable [28]. After averaging many sets of experimental data, the results of simulated glaze icing load experiments were created.

## 4. Results and Discussion

### 4.1. Temperature Effect Experiments

**Figure 6:**
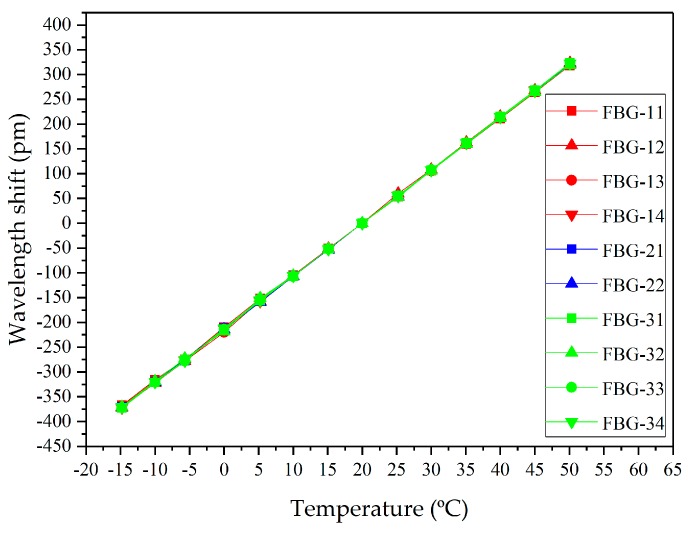
The relationship between temperature and wavelength shift of FBG embedded in the composite insulator.

The results of temperature effect experiments are shown in Figure 6. The wavelength shift curves of ten FBGs are almost overlapping. In other words, the FBG temperature compensation sensor could compensate the wavelength shifts of FBG strain sensors for temperature changes. Besides, the wavelength shifts of all FBGs that were distributed in different places were almost uniform with the temperature changes. Therefore, embedding one FBG temperature compensation sensor into an insulator could effectively eliminate the temperature cross-sensitization.

More specifically, the center wavelength shifts of FBGs were linearly related to environmental temperature, and the goodness of fit was more than 0.997. The straight slopes were the temperature sensitivity whose value was 10.68 pm/°C. Notice that FBG-23 and FBG-24 were broken when they were pasted on the silicone rubber layer, so there was no experimental data for FBG-23 and FBG-24.

As analyzed above, the wavelength separation shifts of FBG strain sensors are independent of the temperature but proportional to the strain generated by glaze icing load via the ceramic tube packing method.

### 4.2. Simulated Glaze Icing Load Experiments

**Figure 7:**
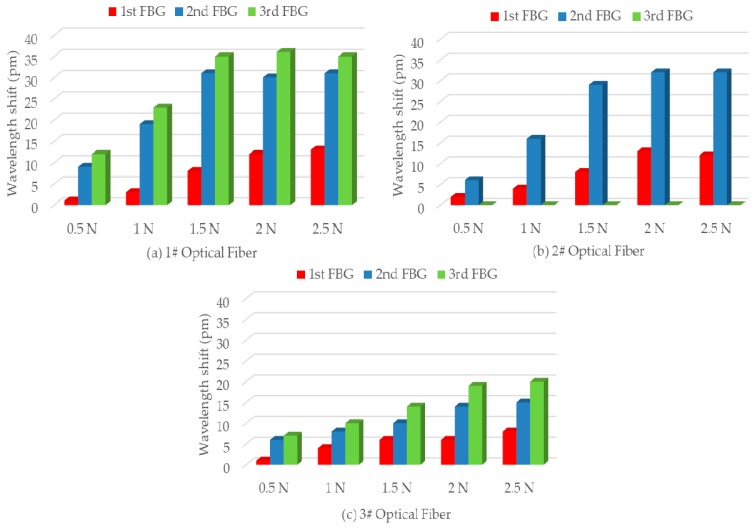
The relationship between wavelength shifts of FBG strain sensors and simulated glaze icing load. (**a**) The wavelength shifts of FBG strain sensors in 1# Optical Fiber; (**b**) The wavelength shifts of FBG strain sensors in 2# Optical Fiber; (**c**) The wavelength shifts of FBG strain sensors in 3# Optical Fiber.

For simulating FBG wavelength shifts when glaze icing load was generated on an insulator shed, simulated glaze icing load experiments were performed by hanging weights. In these experiments, wavelength shifts of an FBG strain sensor were recorded when a weight was hung on the insulator shed edge facing the FBG strain sensor. More specifically, the wavelength shift of FBG-11 was measured when a weight was hung on the 1st big insulator shed edge facing FBG-11. Similarly, the wavelength shift of FBG-12 was measured when a weight was hung on the 1st small insulator shed edge facing FBG-12. The results from other FBG strain sensors were measured in the same way, which are shown in Figure 7.

Figure 7a shows the wavelength shifts of FBG strain sensors in 1# Optical Fiber and compared their strain state in the same simulated glaze icing load. As can be observed, when the same simulated glaze icing load was generated on the insulator shed, the wavelength shift of FBG-11 located in the big insulator shed was less than those of FBG-12 and FBG-13 located in the small insulator sheds. In addition, when low simulated glaze icing load of 0.5 N was generated on the insulator shed, the wavelength shift of FBG-11 was almost 0 pm, whereas the wavelength shifts of FBG-12 and FBG-13 were 9 and 12 pm, respectively. Similarly, FBG strain sensors in 2# Optical Fiber and 3# Optical Fiber have the same relationship with simulated glaze icing load. It can be concluded that the deformation of small insulator sheds was larger than that of big insulator shed in the same glaze icing load, and the FBG strain sensors located in the small insulator sheds were more sensitive than those located in the large insulator sheds. 

Besides that, for FBG-12 and FBG-13 located in 1st and 2nd small insulator shed respectively, when the same simulated glaze icing load was generated on the small insulator shed, the wavelength shift of FBG-13 was slightly higher than that of FBG-12. According to Table 3, the center wavelength of FBG-13 is higher than that of FBG-12. Then, from Equation (2), the strain sensitivity of FBG-13 is higher than that of FBG-12. Thus, the wavelength shift of FBG-13 is much higher in the same simulated glaze icing load. Similarly, FBG strain sensors in 3# Optical Fiber have the same law.

**Figure 8:**
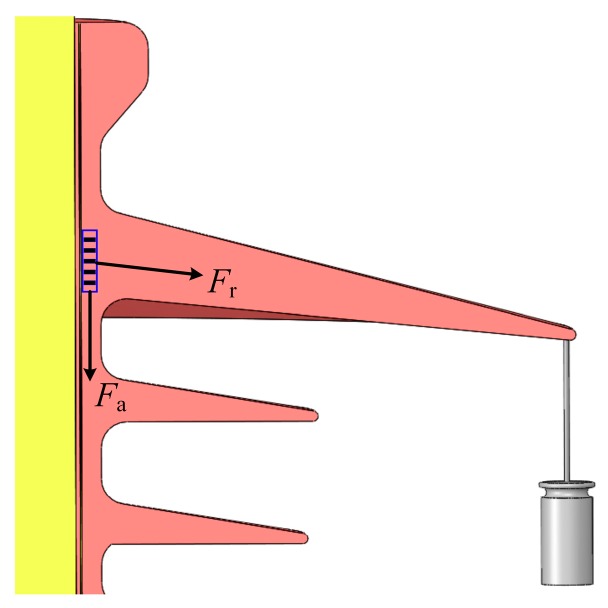
Force analysis on FBG during the simulated glaze icing load experiment.

Figure 7a,b respectively show the wavelength shifts of FBG strain sensors in 1# Optical Fiber and 2# Optical Fiber. With the increase of simulated glaze icing load, the wavelength shifts of FBG-1*n* and FBG-2*n* appear to increase at first, and then remain stable. Specifically, when the simulated glaze icing load reaches a specific value—that was 1.5 N in this paper—the wavelength shifts of FBG strain sensors remain stable. This is because FBG strain sensors were located in the root of insulator sheds. The FBG strain sensors were not only under an axial force, *F*_a_, but also under a radial force, *F*_r_, along the insulator shed radius, as depicted in Figure 8.

Figure 7c shows the wavelength shifts of FBG strain sensors in 3# Optical Fiber. With the increase of simulated glaze icing load, the wavelength shifts of FBG-3*n* gradually increase but are not linearly related to glaze icing load. In addition, the wavelength shifts of FBG-3*n* packed in PVC protection sleeves are less than those of FBG-1*n* and FBG-2*n* in the same simulated glaze icing load. It can be concluded that the protection sleeve weakens the mechanical transmission characteristic from insulator sheds to FBG strain sensors, and reduces the strain sensitivity of FBG strain sensors.

### 4.3. Strain Distribution in Simulated Glaze Icing Load Experiments

**Figure 9:**
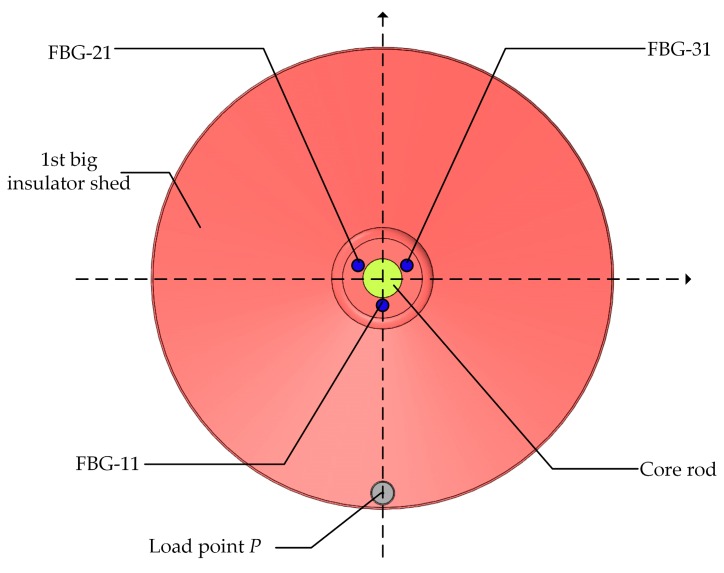
The position of load point *P* in the simulated glaze icing load experiments.

**Figure 10:**
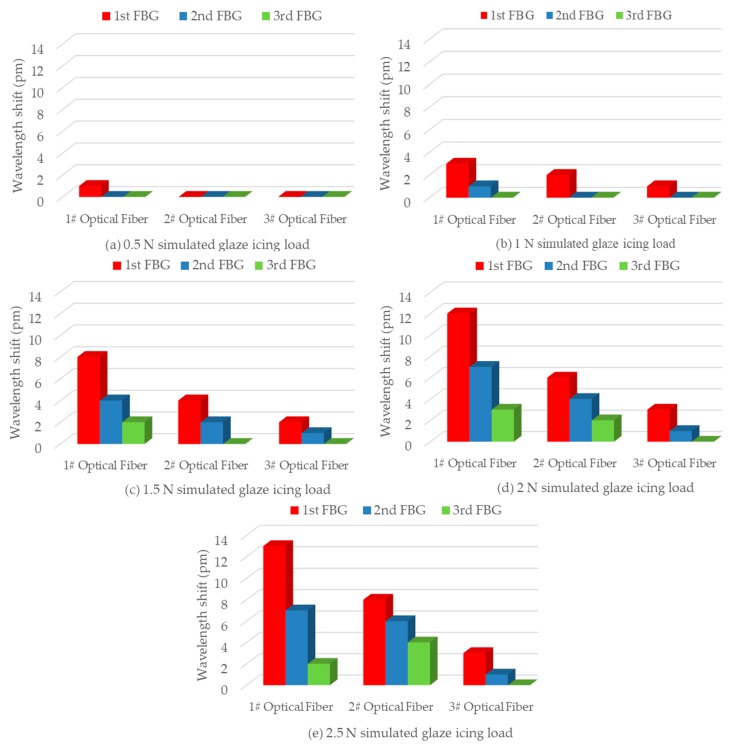
The wavelength shifts of FBG strain sensors when different simulated glaze icing loads were only generated on the first big insulator shed facing FBG11 (*P* point).

To analyze the wavelength shifts of all FBG strain sensors when glaze icing load was generated on a specific insulator shed, a weight was hung on the edge of 1st big insulator shed facing FBG-11, which was labeled as *P* point (Figure 9). The results are shown in Figure 10, which indicates the relationship between wavelength shifts of all FBG strain sensors and the simulated glaze icing load generated on the *P* point. As can be observed, the simulated glaze icing load caused the wavelength shifts of all FBG strain sensors. More specifically, the results show that the whole insulator produces strain distribution when glaze icing load was generated on a specific insulator shed.

For FBG strain sensors in 1# Optical Fiber, under the low simulated glaze icing load of 0.5 N, the wavelength shift of FBG-11 was 1 pm, whereas the wavelength shifts of FBG-12 and FBG-13 were almost 0 pm. The reason is that the deformation of the 1st big insulator shed is small under a low glaze icing load. Also, the strain transfer to FBG-11—which is located 48 mm away from *P* point—was small. In particular, because FBG-12 and FBG-13 were located several shed spacings away from *P* point, the strain of FBG-12 and FBG-13 is much smaller. In addition, under the simulated glaze icing load of 1.5 N, the strain of FBG-12 and FBG-13 could be detected, and the wavelength shift of FBG-12 was 2 pm larger than that of FBG-13 because the position of FBG-12 was closer to *P* point. It can be concluded that the strain transfer decreased with the distance from the load point.

For FBG-11, FBG-21, and FBG-31—which are symmetrically distributed in the 1st big insulator shed with 120° intervals (Figure 10)—under the low simulated glaze icing load of 0.5 N, the wavelength shift of FBG-11 was 1 pm, whereas the wavelength shifts of FBG-21 and FBG-31 were almost 0 pm. The reason is that the deformation of the 1st big insulator shed, with the exception of *P* point, was too small to produce strain on FBG-21 and FBG-31 under a low glaze icing load. Besides that, with the increase of simulated glaze icing load, the wavelength shifts of FBG-21 and FBG-31 gradually increased but were smaller than that of FBG-11. This is because FBG-11 is closest to *P* point, and the strain of FBG-11 facing the load point was maximum, whereas the strain decreases when it transfers to FBG-21 and FBG-31.

Besides this, no matter how much simulated glaze icing load was generated on insulator shed, the wavelength shift of FBG-21 was always larger than that of FBG-31 packed in a PVC protection sleeve. The results indicate that the packaging materials weaken the mechanical transmission characteristic from insulator sheds to FBG strain sensors, and using bare optical fibers was more suitable for detecting glaze icing load.

For FBG strain sensors in 2# and 3# Optical Fibers, the wavelengths of other FBG strain sensors in the same optical fiber began to show shifts when the wavelength shifts of FBG-21 and FBG-31 were more than 2 pm, caused by the simulated glaze icing load of 1.5 N on *P* point. The results indicate that the strain transfer decreased with the distance from the load point *P.*

Above all, when a large enough glaze icing load (i.e., 1.5 N) is generated on an insulator shed, shifts can be observed in the wavelength of FBG strain sensors in the same optical fiber. In particular, the wavelength shift of the FBG strain sensor which is closest to the load point is maximum. Therefore, if the FBG spacing is less than the small insulator shed spacing (i.e., 25 mm), FBG strain sensors in the same optical fiber can detect the strain distribution of the whole insulator when glaze icing load causes deformation of an insulator shed.

## 5. Conclusions

In this paper, a novel method is first proposed to detect glaze icing load of 10 kV composite insulators. Specifically, four FBGs were written in every optical fiber, and three optical fibers were embedded vertically into the composite insulator with even distribution around the central axis. Temperature effect experiments and simulated glaze icing load experiments were performed for verifying the feasibility of the proposed detection method. 

The results showed that the FBG packed in a ceramic tube could solve strain and temperature cross-sensitivity. In particular, the temperature sensitivity average value of FBGs was 10.68 pm/°C. Moreover, it can be concluded that the proposed method could detect glaze icing load of the composite insulator greater than 0.5 N (i.e., 15% of icicle bridged degree). In addition, if the FBG spacing was less than 25 mm, FBG strain sensors in the same optical fiber could detect strain distribution of the whole insulator when glaze icing load was more than 1.5 N. Therefore, the glaze icing load of composite insulators can be detected by using FBGs in the laboratory.

Nevertheless, the proposed method was performed in the laboratory. Thus, the effect of outdoor elements (e.g., sunlight, wind, rain, etc.) were not considered. Hopefully, in further research, embedded FBGs will be located between two sheds for reducing radial force. Future research should also consider the potential effects of non-uniform temperature more carefully. In addition, there is a need for mathematical modeling of strain distribution caused by multiple symmetric glaze icing loads on insulator sheds.

## Figures and Tables

**Table 1:** Optical fiber and FBG parameters.

Description	Values
Diameter of fiber core	10 μm
Diameter of cladding	125 μm
Center wavelength range	1510–1590 nm
Length of FBG	10 mm
Minimum FBG space	10 mm
Reflectivity	≥90%
Side mode suppression ratio	≥10 dB
Working temperature	−40~300 °C

**Table 2:** Parameters of composite insulator FXBW-10/70.

Description		Values
Insulator type		FXBW-10/70
Structural height		373 mm
Insulation height		222 mm
Leakage distance		762 mm
Diameter of the core rod		18 mm
The big insulator shed	Shed overhang	48 mm
	Shed spacing	75 mm
	Number	2
The small insulator shed	Shed overhang	24 mm
	Shed spacing	25 mm
	Number	4

**Table 3:** The initial center wavelengths of FBGs.

Labels of Optical Fibers	Labels of Gratings	Initial Center Wavelengths
1# Optical Fiber	FBG-11	1531.948 nm
	FBG-12	1538.873 nm
	FBG-13	1545.955 nm
	FBG-14	1553.113 nm
2# Optical Fiber	FBG-21	1531.853 nm
	FBG-22	1539.030 nm
	FBG-23	1546.079 nm
	FBG-24	1553.077 nm
3# Optical Fiber	FBG-31	1532.035 nm
	FBG-32	1539.130 nm
	FBG-33	1545.836 nm
	FBG-34	1552.911 nm

**Table 4:** Simulating the glaze icing load generated by an icicle with typical geometry [40].

Weights	Corresponding to the Icicle Length *L*	Corresponding to the Icicle Bridged Degree *η*
0.5 N	11 mm	15%
1.0 N	22 mm	29%
1.5 N	33 mm	44%
2.0 N	44 mm	59%
2.5 N	55 mm	73%
